# Protective effects of 18β-glycyrrhetinic acid on *Pasteurella multocida*–induced vascular inflammatory injury in mice

**DOI:** 10.3389/fvets.2024.1515977

**Published:** 2025-01-09

**Authors:** Qirong Lu, Luyao Wang, Xueping Jiang, Wantong Han, Pu Guo, Yu Liu, Shulin Fu, Jianglin Xiong, Zhongyuan Wu, Yinsheng Qiu

**Affiliations:** ^1^Hubei Key Laboratory of Animal Nutrition and Feed Science, School of Animal Science and Nutritional Engineering, Wuhan Polytechnic University, Wuhan, China; ^2^Wuhan Engineering and Technology Research Center of Animal Disease-Resistant Nutrition, School of Animal Science and Nutritional Engineering, Wuhan Polytechnic University, Wuhan, China

**Keywords:** 18β-glycyrrhetinic acid, inflammatory response, *Pasteurella multocida*, protective effects, vascular injury

## Abstract

*Pasteurella multocida* (Pm) is a widespread zoonotic pathogen with the ability to infect wild animals, livestock, and humans. Pm infection can cause haemorrhagic pneumonia, indicating that the pathogenesis involves serious vascular injury and inflammation. 18β-Glycyrrhetinic acid (GA) has cardiovascular protective and anti-inflammatory effects, but its effect on vascular injury caused by Pm infection is not clear. This study focused on the protective effects of GA on Pm-induced vascular inflammatory injury in mice. The results showed that GA intervention significantly improved the survival rate and the changes in haematological and biochemical parameters caused by Pm infection in mice. Haematoxylin and eosin staining revealed that GA delayed the progression of vascular injury, including abnormalities in elastic fibres, local rupture of the vascular intima, and inflammatory cell infiltration in response to Pm infection. The immunohistochemical results showed that after the GA intervention, the vascular inflammatory response in Pm-infected mice was alleviated. These protective effects may be related to the reduced expression of poly (ADP-ribose) polymerase-1, high mobility group box 1, interleukin-1β, and interleukin-18 in vascular tissue by GA. These findings suggest that GA inhibits the activation of inflammation to protect vascular injury *in vivo*. Hence, GA exhibits therapeutic potential in the treatment of vascular injury.

## 1 Introduction

*Pasteurella multocida* (Pm) is an opportunistic zoonotic pathogen that has the ability to infect a diverse range of hosts, including wild and domestic animals, and humans (1–3). Pm can be categorised into five groups (A, B, D, E and F) based on the bacterial capsular characteristics (4). Among them, capsular type A mainly causes porcine pneumonia, and can also cause septicaemia and death of pigs in serious cases (5, 6). Pm infection is characterised by acute respiratory disease accompanied by hyperthermia, dyspnoea, and death within a few days. The primary lesions observed upon postmortem examination are suppurative bronchopneumonia, necrohaemorrhagic fibrinous pleuropneumonia, fibrinous pericarditis, and peritonitis (7). Haemorrhagic pneumonia caused by Pm infection involves necrosis of the vessel walls (7) and severe damage to the vascular endothelium with increased permeability (8), suggesting that there the pathogenesis involves serious vascular injury and inflammation. Healthy vascular endothelial function can provide an antioxidant, anti-inflammatory, and antithrombotic interface for the body, which plays a vital role in maintaining the health and homeostasis of multiple organs (9). The vascular response is the central link of the inflammatory process and is the key to the occurrence and development of infectious systemic inflammatory response (10–12). At present, antibiotics and vaccines are mainly used to treat and prevent Pm infection in veterinary medicine (13–16), but they cannot solve the problem of vascular inflammatory injury caused by Pm infection. Therefore, it is urgent to seek candidate drugs to control the vascular inflammatory injury associated with Pm infection in pigs.

In traditional medicine, liquorice is used to treat respiratory diseases, hyperphagia, fever, gastric ulcer, rheumatism, skin diseases, haemorrhagic diseases, and jaundice (17). 18β-Glycyrrhetinic acid (GA), a major active component obtained from liquorice, shows a broad range of biological properties, including antibacterial, anti-inflammatory, antiviral, antioxidant, antiasthmatic, and anticancer activities, and hepatoprotective and cardioprotective effects (18, 19). Researchers have shown that GA can alleviate vascular injury caused by various factors. GA administration significantly improved the pathological changes in the pulmonary vasculature induced by monocrotaline in rats (20). Moreover, GA alleviated endoplasmic reticulum stress–induced inflammation in pulmonary arterial hypertension (21) and pulmonary artery injuries in rats with high-altitude pulmonary hypertension (22). In addition, poly (ADP-ribose) polymerase-1 (PARP1) is closely related to vascular endothelial dysfunction and the activation of inflammatory cells (23). In our previous study, we demonstrated that GA could alleviate Pm-induced vascular endothelial inflammatory injury by PARP1-mediated nuclear factor kappa B (NF-κB) and high mobility group box 1 (HMGB1) signalling suppression in porcine vascular endothelial cells (PIECs) (8). These studies have demonstrated that GA has beneficial effects on vascular inflammatory injury. However, whether it also protects against vascular inflammatory injury caused by Pm infection *in vivo* remains to be investigated.

The purpose of this study was to investigate the protective effects of GA on Pm-induced vascular inflammatory injury *in vivo*, and to provide additional evidence that GA could be used as a new antibiotic substitute or feed additive for adjuvant treatment of inflammatory injury. The results provide a theoretical basis for clinical relief of vascular inflammatory injury caused by Pm infection.

## 2 Materials and methods

### 2.1 Reagents and chemicals

GA (CAS No.: 471–53-4) was purchased from MedChemExpress (USA) and Dimethyl sulfoxide (CAS No.: 67-68-5) was purchased from Sigma-Aldrich (Germany).

### 2.2 Ethics statement

All animal experiments were approved by the Animal Ethics Committee of Wuhan Polytechnic University under permit number WPU202311004.

### 2.3 Bacterial strain culture

The Pm HB03 strain was kindly provided by Prof. Bin Wu (Huazhong Agricultural University, Wuhan, China) and cultured in Tryptose Soya Broth (Hopebio, China) with 5% (v/v) Newborn Calf Serum (Tianhang, China) at 37°C.

### 2.4 Animal experiment design

Specific pathogen–free female BALB/c mice (20–25 g in weight) were purchased from the Center of Laboratory Animals of Hubei Province, Wuhan, China. During the experiment, the temperature of the animal room was 22 ± 1°C, and the mice had free access to food and water. To avoid stress in mice, they were acclimatised for 3 days and then randomly divided into five groups, followed by drug treatment and Pm challenge. There five groups were: control (*n* = 10), Pm challenge (*n* = 10), 10 mg/kg body weight (b.w.) GA intervention (*n* = 10), 20 mg/kg b.w. GA intervention (*n* = 10) and 40 mg/kg b.w. GA intervention (*n* = 10). The infection was established by intraperitoneal injection of Pm. The challenge dose was 100 μL of 80 colony-forming units of Pm HB03 (24); the control group was injected with an equivalent volume of phosphate-buffered saline (PBS). The intervention group was injected with GA solution (10, 20 and 40 mg/kg b.w.) intramuscularly, and the control and challenge groups were injected with the same volume of PBS. The mice were administered GA for the first time on the day of challenge, 2 h before the Pm challenge, and then once a day for 5 consecutive days. The survival rate was assessed daily up to 5 days. Prior to euthanasia, the mice were fasted overnight.

### 2.5 Haematological and biochemical parameters

Blood samples were collected from the mice at sacrifice to analyse haematological and serum biochemical parameters. The haematological parameters were determined using a BC-5000 vet Hematology Analyzer (Mindray, China). The following haematological parameters were determined: the number of white blood cells (WBC), neutrophils (Neu), lymphocytes (Lym), and eosinophils (Eos). For the biochemical parameters, heparinised blood samples were centrifuged for 10 min at 3500 rpm and 4°C; then, the supernatant was collected. An automatic blood biochemical analyzer (HITACHI 7100; Hitachi, Tokyo, Japan) was used to determine total bilirubin (TBIL), total protein (TP), albumin (ALB), aspartate aminotransferase (AST) activity, alanine aminotransferase (ALT) activity, triglycerides (TG), glucose (GLU), and creatinine (CRE). All haematological and biochemical parameters were measured by following standard procedures.

### 2.6 Histopathological analysis

After collecting vascular tissue samples from the mice, they were fixed in 4% neutral buffered paraformaldehyde (Servicebio, China), dehydrated through a graded alcohol series, embedded in paraffin wax, and cut into 5-μm sections. The sections were stained with a haematoxylin and eosin staining kit (Baiqiandu, China), and the histopathological changes in the vascular tissues were examined using a microscope with and an imaging system (NIKON, Japan).

### 2.7 Immunohistochemical analysis

The tissue distribution of the PARP1, HMGB1, interleukin-1β (IL-1β), and interleukin-18 (IL-18) proteins was detected using IHC analysis. In brief, paraffin sections of vascular tissues were blocked with 3% bovine serum albumin solution after antigen retrieval. Subsequently, these sections were incubated with a PARP1 polyclonal antibody (Proteintech, China), an HMGB1 polyclonal antibody (Abclonal, China), an IL-1β polyclonal antibody (Abclonal, China), or an IL-18 polyclonal antibody (Abclonal, China) overnight at 4°C. After washing with 1× PBS several times, the sections were incubated with goat anti-rabbit IgG conjugated to horseradish peroxidase (Abclonal, China) for 50 min. Finally, the sections were incubated at room temperature with a 3,3′-diaminobenzidine kit (zsbio, China), counterstained with haematoxylin (Baiqiandu, China), and encapsulated in neutral resin film (Baiqiandu, China) following conventional dewatering. The slides were evaluated using the light microscope with an imaging system (NIKON, Japan).

### 2.8 Statistical analysis

The results are presented as mean ± standard deviation. SPSS Statistics 18.0 for Windows (SPSS Inc., Chicago, IL, United States) was used for statistical analysis. Statistical significance was accepted for *p* < 0.05.

## 3 Results

### 3.1 Effect of GA on the survival rate of Pm-infected mice

**Figure 1:**
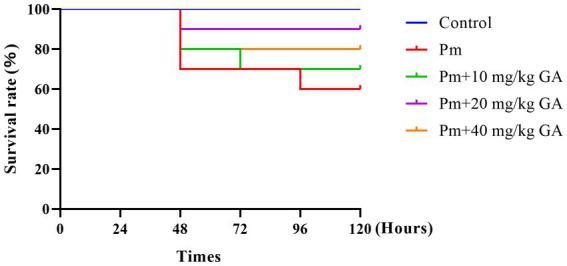
The effect of GA on the survival rate of Pm-infected mice.

To study the protective effect of GA on Pm infection, we established a mouse model of Pm infection. Compared with the control group, the Pm challenge group showed symptoms of listlessness, crouching, anorexia, and depression, and their fur began to lose its lustre. The GA intervention groups showed marked improvement in these symptoms. Meanwhile, Figure 1 shows the survival rate of the mice. The survival rate on day 5 was lower in the Pm challenge and GA intervention groups compared with the control group, but the GA intervention groups showed a higher survival rate compared with the Pm challenge group. This suggests that GA alleviates Pm infection–induced death.

### 3.2 Effect of GA on Pm-induced haematological and biochemical parameters

Table 1 shows the results of the haematological analysis. Compared with the control group, the Pm challenge group showed a significant increase in the number of WBC, Neu, and Eos, and a significant decrease in the number of Lym. Compared with the Pm challenge group, the 20 mg/kg b.w. GA intervention group showed a significant decrease in the number of WBC, and the 40 mg/kg b.w. GA intervention group showed a significant decrease in the number of WBC and Neu. The results indicated that Pm-infected mice developed an inflammatory injury, and GA could alleviate this abnormality.

**Table 1:** The effect of GA on haematological parameters in Pm-infected mice.

Parameters	Group A	Group B	Group C	Group D	Group E	*p* value			
						Group B vs. Group A	Group B vs. Group C	Group B vs. Group D	Group B vs. Group E
WBC (10^^9^/L)	6.62 ± 0.85	13.28 ± 3.09	12.42 ± 1.25	11.10 ± 0.38	8.30 ± 1.45	<0.01	0.42	<0.05	<0.01
Neu (10^^9^/L)	0.74 ± 0.08	6.97 ± 3.00	5.32 ± 2.07	5.26 ± 1.94	3.31 ± 0.80	<0.01	0.12	0.14	<0.01
Lym (10^^9^/L)	5.72 ± 1.01	2.08 ± 0.41	2.33 ± 1.04	2.18 ± 0.27	2.08 ± 0.51	<0.01	0.45	0.77	1.00
Mon (10^^9^/L)	0.24 ± 0.08	0.41 ± 0.30	0.35 ± 0.21	0.30 ± 0.07	0.37 ± 0.20	0.07	0.06	0.33	0.69
Eos (10^^9^/L)	0.08 ± 0.04	0.32 ± 0.19	0.50 ± 0.23	0.52 ± 0.23	0.46 ± 0.21	<0.01	<0.05	<0.05	0.10

Group A is the control group; group B is the Pm challenge group; group C is the 10 mg/kg b.w. GA intervention group; group D is the 20 mg/kg b.w. GA intervention group; and group E is the 40 mg/kg b.w. GA intervention group. *p* < 0.05 was defined as significant difference, *p* < 0.01 was defined as extremely significant difference.

Table 2 shows the results of the biochemical analysis. Compared with the control group, the TBIL, AST, CRE, TP and ALT levels were significantly increased in the Pm challenge group, while the ALB and GLU levels were significantly decreased. Compared with the Pm challenge group, the 20 mg/kg b.w. GA intervention group showed a significant decrease in the ALT level and a significant increase in the GLU and ALB levels; the 40 mg/kg b.w. GA intervention group showed a significant decrease in the CRE and ALT levels. The results showed that Pm infection caused liver and kidney dysfunction and abnormal glucose metabolism, and GA could alleviate these abnormalities.

**Table 2:** The effect of GA on the biochemical parameters in Pm-infected mice.

Parameters	Group A	Group B	Group C	Group D	Group E	*p* value			
						Group B vs. Group A	Group B vs. Group C	Group B vs. Group D	Group B vs. Group E
TBIL (mg/dl)	0.52 ± 0.17	1.13 ± 0.07	0.82 ± 0.14	0.74 ± 0.30	0.72 ± 0.56	<0.01	0.18	0.07	0.17
TP (g/dL)	37.01 ± 8.69	49.12 ± 4.55	48.37 ± 3.87	46.96 ± 3.54	44.39 ± 19.3	<0.05	0.91	0.75	0.42
ALB (g/dL)	27.47 ± 5.08	17.15 ± 2.94	20.22 ± 2.04	20.00 ± 2.09	25.83 ± 12.78	<0.01	0.33	0.39	<0.05
AST (U/L)	102.64 ± 20.21	178.44 ± 103.84	166.8 ± 22.31	152.8 ± 29.32	153.33 ± 47	<0.01	0.73	0.45	0.534
ALT (U/L)	29.87 ± 8.04	55.25 ± 44.71	40.83 ± 18.00	26.80 ± 11.43	20.71 ± 27.20	<0.05	0.27	<0.05	<0.05
GLU (mg/dL)	3.87 ± 0.37	1.40 ± 0.29	2.07 ± 0.99	2.24 ± 0.69	2.50 ± 1.25	<0.01	0.07	0.03	<0.05
CRE (mg/dL)	10.93 ± 5.07	31.93 ± 2.44	29.49 ± 6.93	27.44 ± 4.05	22.66 ± 7.89	<0.01	0.45	0.19	<0.05

Group A is the control group; group B is the Pm challenge group; group C is the 10 mg/kg b.w. GA intervention group; group D is the 20 mg/kg b.w. GA intervention group; and group E is the 40 mg/kg b.w. GA intervention group. *p* < 0.05 was defined as significant difference, *p* < 0.01 was defined as extremely significant difference.

### 3.3 Effect of GA on Pm-induced vascular histopathology

**Figure 2:**
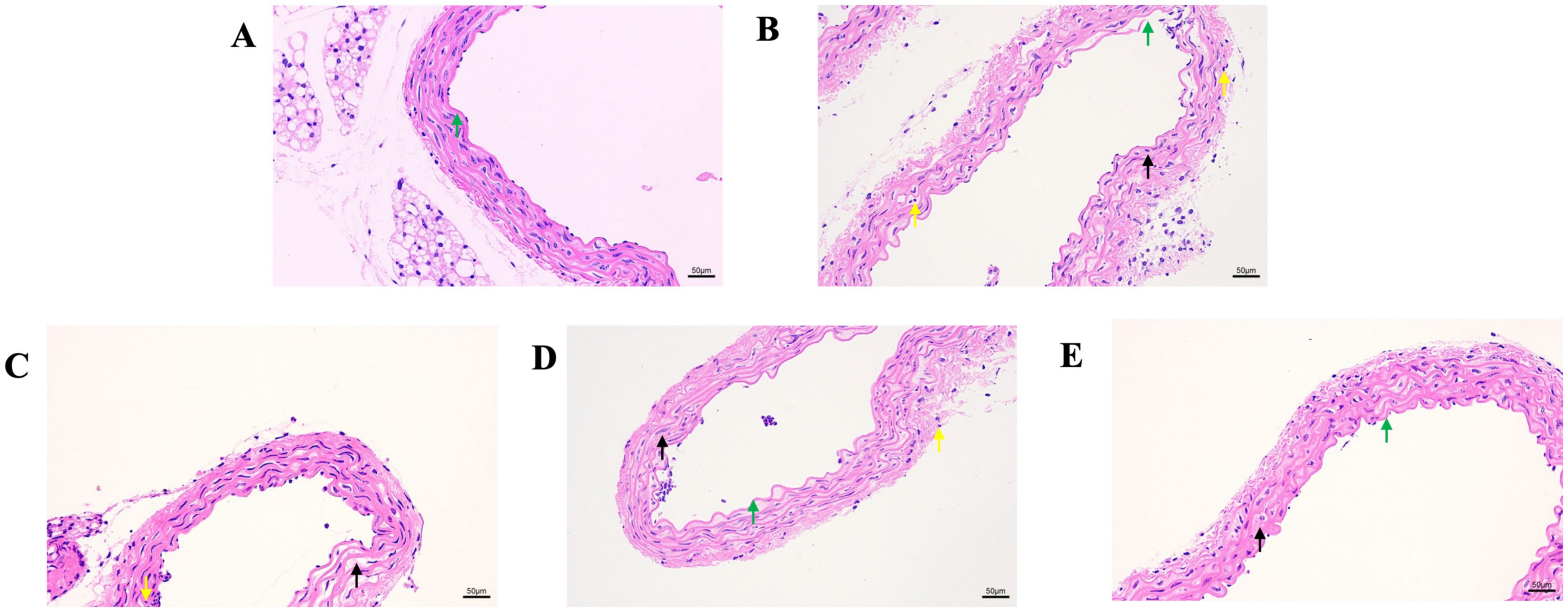
The effect of GA on vascular pathological changes in Pm-infected mice. Bar = 50 μm **(A)** Control group; **(B)** Pm infection group; **(C)** 10 mg/kg b.w. GA intervention group; **(D)** 20 mg/kg b.w. GA intervention group; **(E)** 40 mg/kg b.w. GA intervention group.

Next, we evaluated the effect of GA on the Pm-induced histopathological changes in the blood vessels (Figure 2). The control showed a normal vascular structure (Figure 2A). Compared with the control group, the Pm challenge group showed some unclear elastic fibres that were disordered and partially broken (Figure 2B, black arrow), local rupture of the vascular intima (Figure 2B, green arrow) and a large number of inflammatory cells (Figure 2B, yellow arrow). Compared with the Pm challenge group, the GA intervention groups showed fewer inflammatory cells in the blood vessels and a reduction in the structural damage to the elastic fibres (Figures 2C–E). The results showed that GA could improve vascular inflammatory injury caused by Pm infection.

### 3.4 GA inhibits the vascular inflammatory injury caused by Pm infection

**Figure 3:**
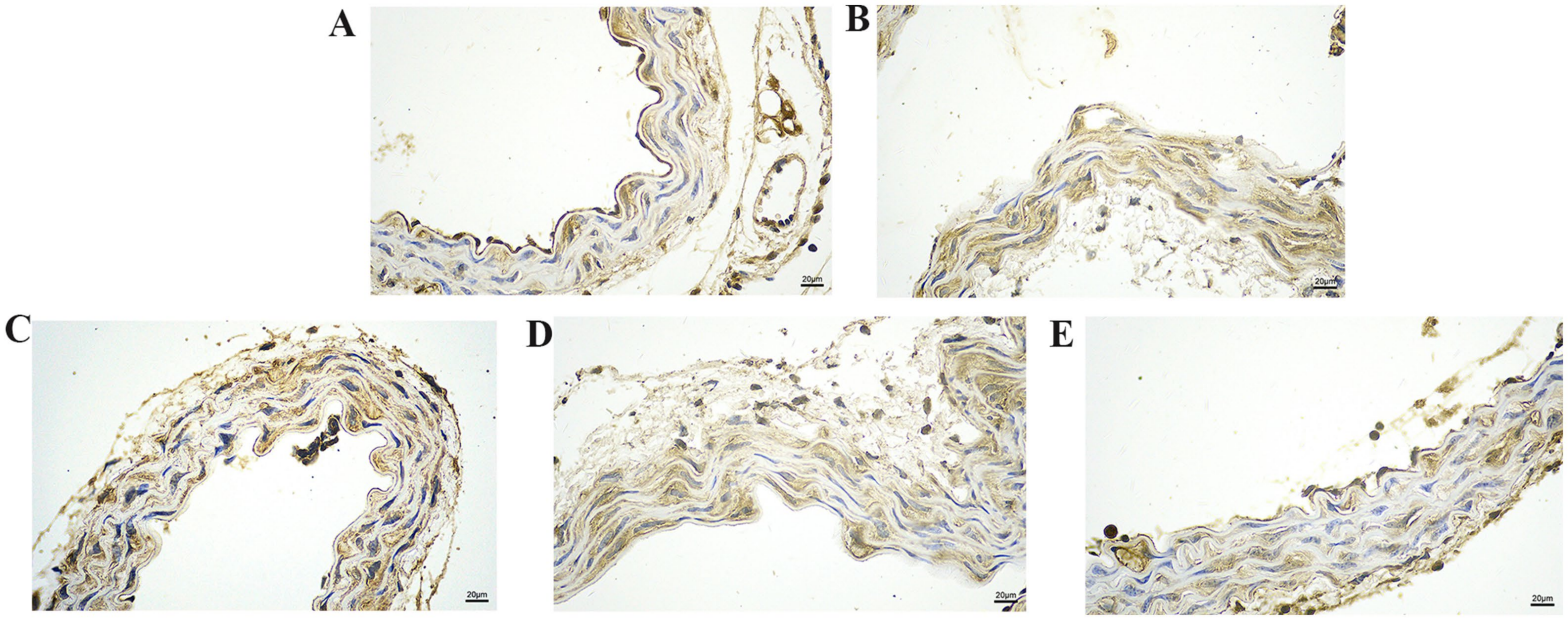
The effect of GA on PARP1 protein expression in vascular tissue from Pm-infected mice. Bar = 20 μm **(A)** Control group; **(B)** Pm infection group; **(C)** 10 mg/kg b.w. GA intervention group; **(D)** 20 mg/kg b.w. GA intervention group; **(E)** 40 mg/kg b.w. GA intervention group.

**Figure 4:**
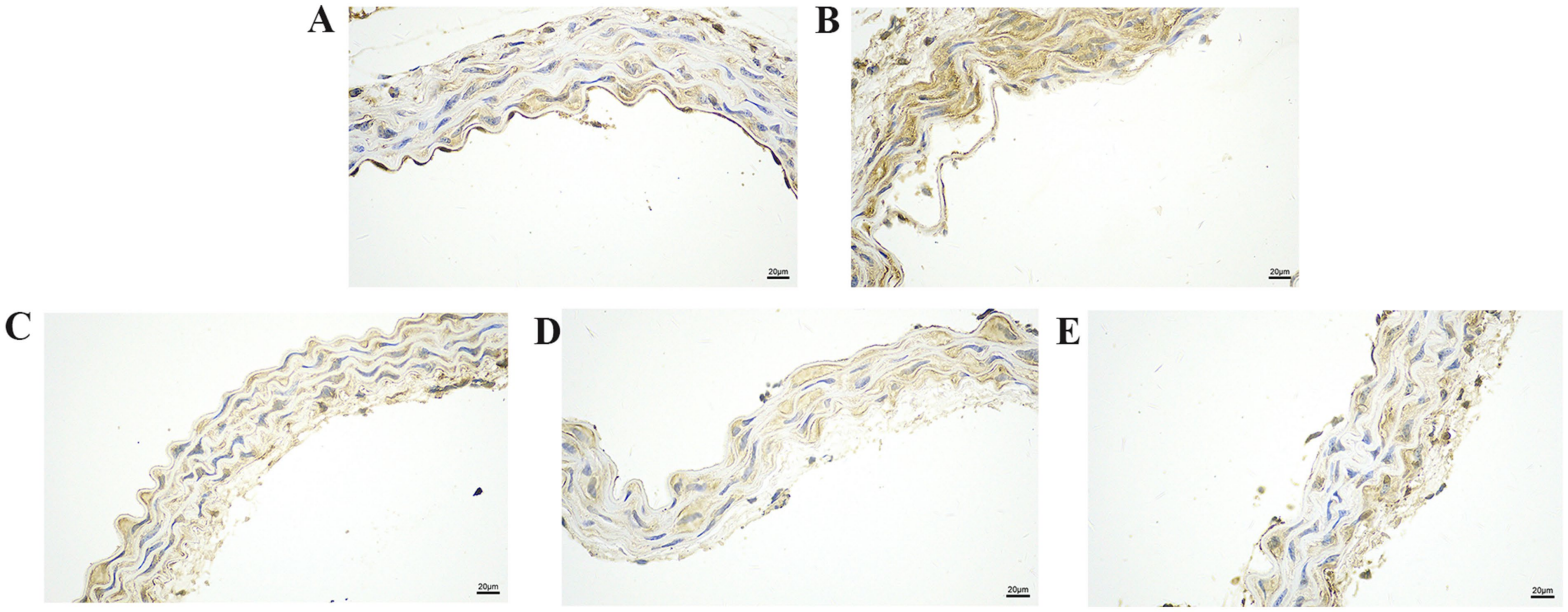
The effect of GA on HMGB1 protein expression in vascular tissue from Pm-infected mice. Bar = 20 μm **(A)** Control group; **(B)** Pm infection group; **(C)** 10 mg/kg b.w. GA intervention group; **(D)** 20 mg/kg b.w. GA intervention group; **(E)** 40 mg/kg b.w. GA intervention group.

**Figure 5:**
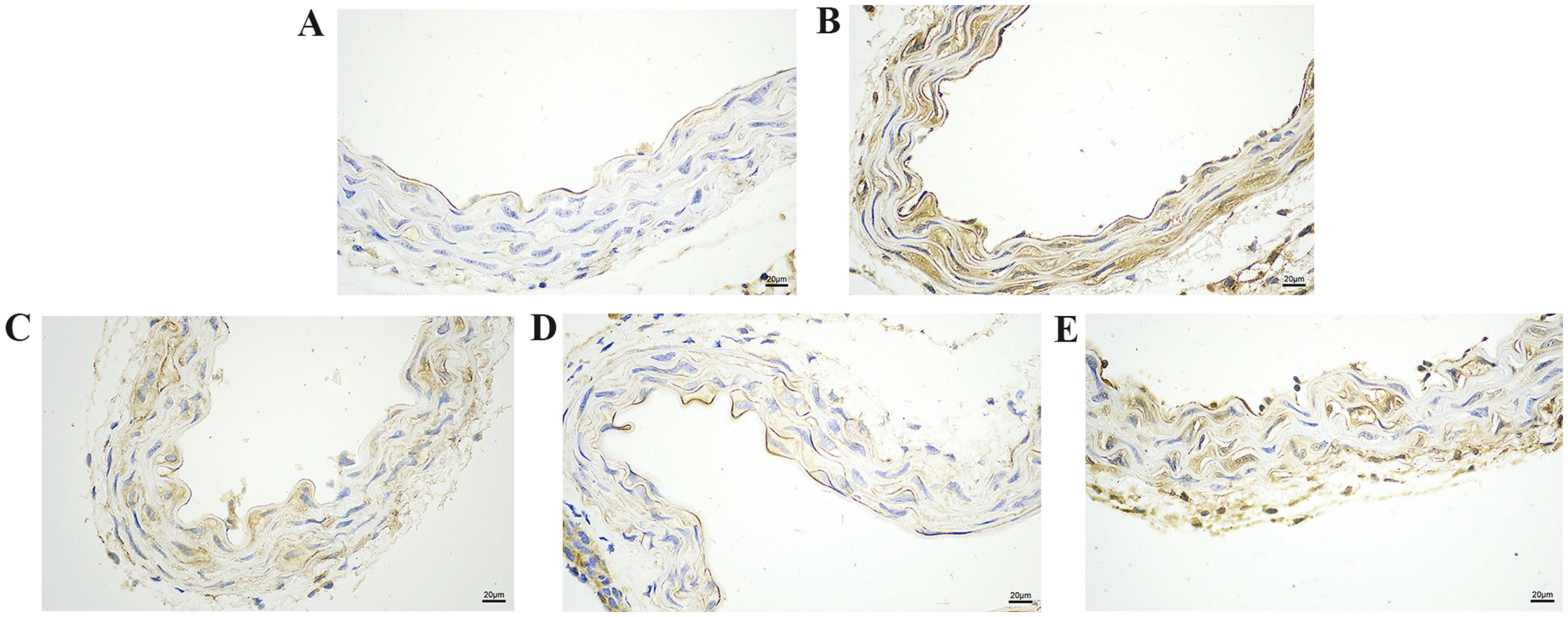
The effect of GA on IL-1β protein expression in vascular tissue from Pm-infected mice. Bar = 20 μm **(A)** Control group; **(B)** Pm infection group; **(C)** 10 mg/kg b.w. GA intervention group; **(D)** 20 mg/kg b.w. GA intervention group; **(E)** 40 mg/kg b.w. GA intervention group.

**Figure 6:**
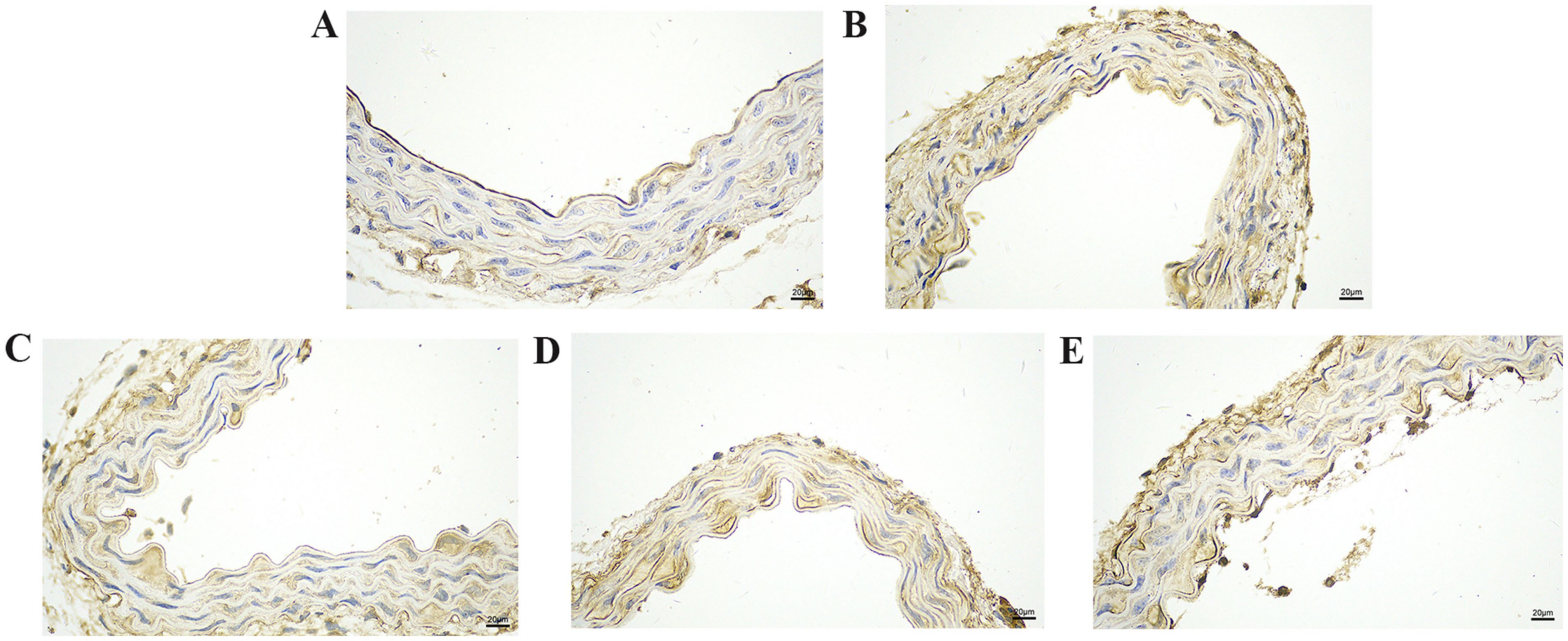
The effect of GA on IL-18 protein expression in vascular tissue from Pm-infected mice. Bar = 20 μm **(A)** Control group; **(B)** Pm infection group; **(C)** 10 mg/kg b.w. GA intervention group; **(D)** 20 mg/kg b.w. GA intervention group; **(E)** 40 mg/kg b.w. GA intervention group.

We further explored the effect of GA on the expression of inflammatory proteins in the blood vessels by performing IHC analysis (Figures 3–6). Compared with the control group, the Pm challenge group showed significantly increased PARP1, HMGB1, IL-1β, and IL-18 protein expression in the blood vessels, indicating that Pm infection in mice triggered vascular inflammatory injury. Compared with the Pm challenge group, the GA intervention groups showed reduced PARP1, HMGB1, IL-1β and IL-18 protein levels in vascular tissue to varying degrees depending on the GA dose. Thus, GA had a protective effect on vascular inflammatory injury in Pm-infected mice.

## 4 Discussion

**Figure 7:**
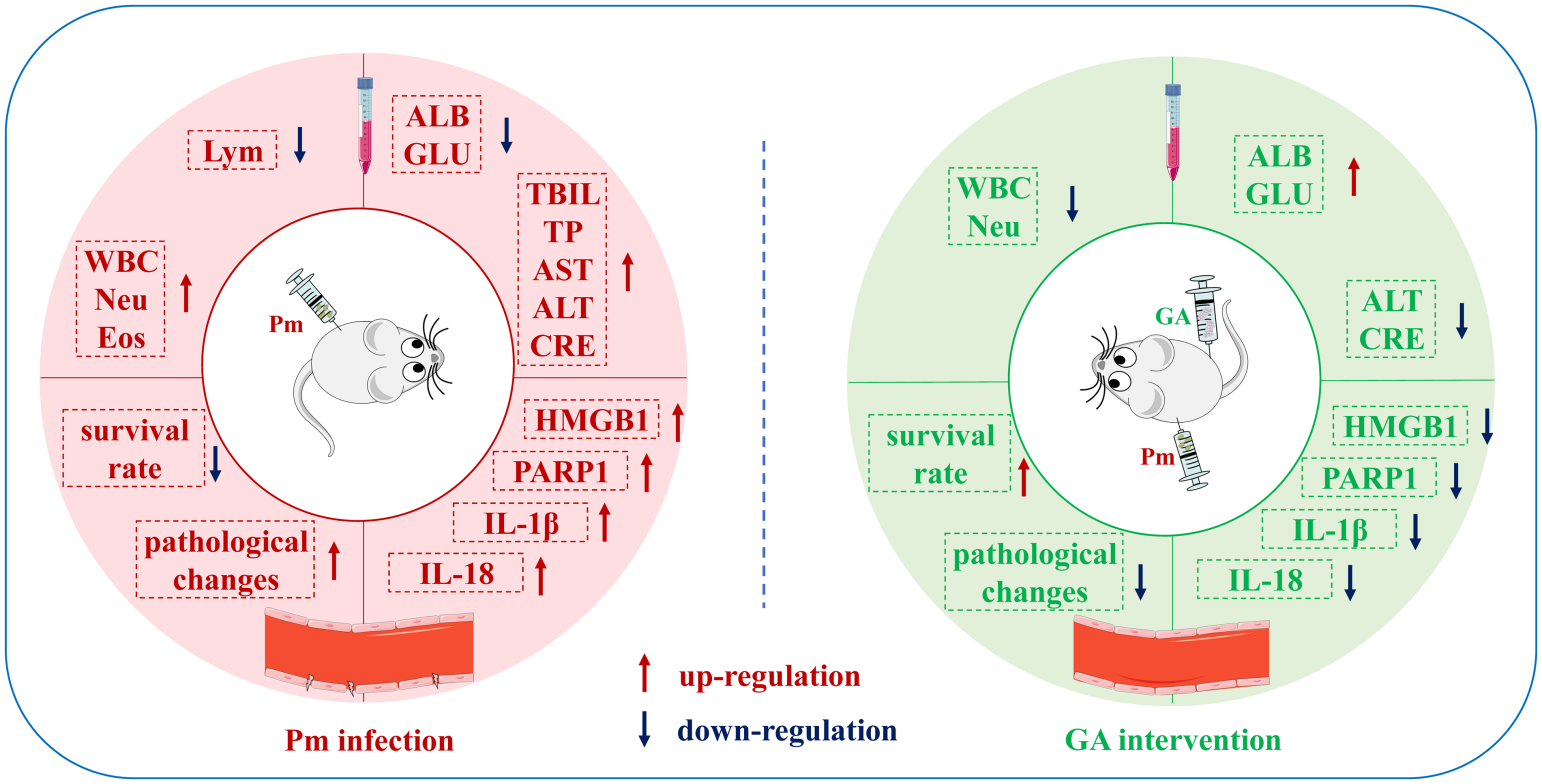
Schematic representation of the proposed protective effects of GA on Pm-induced vascular inflammatory injury in mice. In this study, vascular injury was established in mice via Pm infection, and the ability of GA to reduce this injury was explored. GA could significantly ameliorate the abnormal haematological and biochemical parameters and vascular pathological changes in mice caused by Pm infection, and reduce the expression of vascular inflammatory proteins.

Pm is a gram-negative bacterium that can infect both domestic animals and humans, leading to large economic losses to the livestock industry (25). Haemorrhagic pneumonia caused by Pm infection characterised by the haemorrhagic multifocal areas, necrosis of the vessel walls, and associated with suppurative inflammatory exudation (7), which means that Pm infection can induce vascular inflammatory injury. In veterinary clinical practice, vaccination and antibiotics are mainly used to prevent and treat Pm infection (26). However, this approach cannot solve the problem of vascular inflammatory injury caused by Pm infection, and the widespread illegal use of antibiotics can easily lead to the development of drug resistance (27–29). Moreover, natural products have the potential to serve as candidate drugs, thereby exerting a relieving effect on vascular inflammatory injury (30, 31). Therefore, it is of great clinical significance to study the vascular inflammatory injury caused by Pm infection and the regulatory effects of natural products. In the present study, we used Pm to construct a mouse model of vascular injury, and explored the role of GA in alleviating this vascular inflammatory injury. We found that the GA intervention significantly improved the vascular pathological changes and reduced the expression of vascular inflammatory proteins caused by Pm infection in mice (Figure 7). Elucidating the regulatory role of GA in vascular inflammatory injury will provide a theoretical basis for the prevention and control of Pm infection and the development of alternative antibiotic therapies and feed additives.

Previous studies have shown that GA alleviates the inflammatory injury of PIECs caused by Pm infection (8). Mice are a useful animal model to study Pm infection (32), and to explore whether GA has protective effect on vascular inflammatory injury caused by Pm infection *in vivo*. Mice infected by Pm show a dull coat, depression, anorexia, wrinkles, and reluctance to move, and the lungs are congested and bleed (33). In this study, Pm-infected mice also showed similar clinical symptoms to those of Pm-infected mice, such as appetite loss, messy fur, and depression (32). Compared with the control group, the Pm challenge group presented significantly increased mortality (24, 34), a finding consistent with the fact that the Pm HB03 strain is classified as highly virulent. Natural extracts have therapeutic effects on animals infected by Pm (35). We found that GA, isolated from liquorice, could improve the survival rate of mice compared with the Pm challenge group, demonstrating the potential therapeutic effect of GA.

When a host is infected by a pathogen, the body responds to the invading pathogen, and there are changes in haematological and biochemical parameters. Praveena et al. (36) reported that after Pm infection in mice, the number of Neu increased significantly, the number of Lym decreased significantly, and the TP level in the blood increased, changes that are consistent with the results of the present study. Meanwhile, biomarkers of liver and kidney injury (AST, ALT, and CRE) increase significantly in the Pm-infected mice (37). We noted similar results changes in the blood biochemical parameters in the present study, indicating that Pm infection in mice impairs liver and kidney function. In a previous study, researchers reported elevated serum CRE and lactate dehydrogenase levels in BALB/c mice with renal injury; treatment with 10, 25, or 50 mg/kg b.w. GA significantly decreased these biochemical parameters (38). In the present study, slightly difference GA concentrations alleviated the abnormal haematological and biochemical parameters in mice after Pm infection, suggesting that GA may alleviate the inflammatory reaction in mice caused by Pm infection.

The invasion of a host by pathogenic bacteria can lead to congestion, swelling, and even bleeding at the infection site, accompanied by an inflammatory reaction. Researchers have found that mice infected by Pm show pulmonary congestion, obvious vasodilation, haemorrhage, serum protein exudation, leucocyte recruitment, and other phenomena (33, 39). Pm-infected mice show a marked inflammatory cell infiltration in the alveolar wall and a large number of red blood cells in the alveolar cavity, indicating local haemorrhage (40). Our histopathological examination revealed that the intima of blood vessels in the Pm-infected mice was damaged, accompanied by marked inflammatory cell infiltration. GA could effectively alleviate this vascular inflammatory injury caused by Pm infection. In chickens, the inflammatory factors HMGB1 and IL-6 are widely expressed in the lungs after Pm infection (41). Moreover, bacterial infection can promote the expression of PARP1 and the activation of inflammation related pathways (42, 43), and the inhibition of PARP1 has been repeatedly confirmed by experimental models to improve the inflammatory response and increase the survival rate (44, 45). In addition, our previous study showed that GA could alleviate Pm-induced vascular endothelial inflammation via PARP1-mediated HMGB1 signalling suppression in PIECs (8). Consistent with those prior findings, we found that Pm infection increased PARP1, HMGB1, IL-1β, and IL-18 protein expression in vascular tissue, while GA reduced the expression of these inflammatory factors. These data suggest that GA might ameliorate vascular inflammatory injury in mice via a PARP1–HMGB1 signalling pathway.

In conclusion, we showed that in a mouse model of Pm infection, GA significantly reduced Pm-induced vascular inflammatory injury, and decreased PARP1, HMGB1, IL-1β, and IL-18 protein expression in vascular tissue. PARP1-mediated HMGB1 signalling suppression may represent a critical pathway for treating Pm-induced vascular inflammatory injury. We have provided data that should be useful for preclinical and clinical studies that evaluate the potential of GA to prevent vascular inflammatory injury caused by Pm infection.

## Data Availability

The original contributions presented in the study are included in the article/supplementary material, further inquiries can be directed to the corresponding authors.
